# Advances in cancer immunotherapy 2019 – latest trends

**DOI:** 10.1186/s13046-019-1266-0

**Published:** 2019-06-19

**Authors:** Stephan Kruger, Matthias Ilmer, Sebastian Kobold, Bruno L. Cadilha, Stefan Endres, Steffen Ormanns, Gesa Schuebbe, Bernhard W. Renz, Jan G. D’Haese, Hans Schloesser, Volker Heinemann, Marion Subklewe, Stefan Boeck, Jens Werner, Michael von Bergwelt-Baildon

**Affiliations:** 1 Department of Medicine III, University Hospital Munich, LMU Munich, Marchioninistr. 15, D-81377 Munich, Germany; 2 Department of General, Visceral, and Transplantation Surgery, University Hospital, LMU Munich, Munich, Germany; 3 Center of Integrated Protein Science Munich (CIPS-M) and Division of Clinical Pharmacology, Department of Medicine IV, University Hospital, LMU Munich, Munich, Germany; 4 Institute of Pathology, LMU Munich, Munich, Germany; 5 University Hospital of Cologne, Cologne, Germany; 6 German Cancer Consortium (DKTK), Partner Site Munich; and German Cancer Research Center (DKFZ), Heidelberg, Germany; 7 Center for Molecular Medicine Cologne (CMMC), Cologne, Germany; 8 Gene Center LMU, Munich, Germany

**Keywords:** Immunotherapy, Programmed cell death protein 1 (PD-1), Programmed cell death protein ligand 1 (PD-L1), Chimeric antigen receptor T cells (CAR T cells), Trends, Regional distribution

## Abstract

Immunotherapy has become an established pillar of cancer treatment improving the prognosis of many patients with a broad variety of hematological and solid malignancies. The two main drivers behind this success are checkpoint inhibitors (CPIs) and chimeric antigen receptor (CAR) T cells. This review summarizes seminal findings from clinical and translational studies recently presented or published at important meetings or in top-tier journals, respectively. For checkpoint blockade, current studies focus on combinational approaches, perioperative use, new tumor entities, response prediction, toxicity management and use in special patient populations. Regarding cellular immunotherapy, recent studies confirmed safety and efficacy of CAR T cells in larger cohorts of patients with acute lymphoblastic leukemia or diffuse large B cell lymphoma. Different strategies to translate the striking success of CAR T cells in B cell malignancies to other hematological and solid cancer types are currently under clinical investigation. Regarding the regional distribution of registered clinical immunotherapy trials a shift from PD-1 / PD-L1 trials (mainly performed in the US and Europe) to CAR T cell trials (majority of trials performed in the US and China) can be noted.

## Background

The importance of immunotherapy has been acknowledged by the *Nobel prize for physiology or medicine* 2018 awarded for the discovery of *cytotoxic T-lymphocyte-associated protein* (CTLA-4) to James P. Allison and *programmed cell death protein 1* / *programmed cell death protein ligand 1* (PD-1 / PD-L1) to Tasuku Honjo [1]. Malignant tumors take advantage of the inhibitory PD-1 / PD-L1 or CTLA-4 pathways to evade the immune system [2]. Disruption of this axis by blocking monoclonal antibodies can induce durable remissions in different cancer types and has led to numerous FDA and EMA approvals, among others, for the treatment of melanoma, lung cancer, urothelial cancer, head and neck squamous cell carcinoma (HNSCC), renal cell cancer (RCC) and Hodgkin’s disease [3]. Up-to-date reviews providing a comprehensive overview of approved indications for different CPIs have been published previously [3, 4].

This review focuses on clinical and pre-clinical findings that might guide future clinical application of CPIs in general. We identified potentially trendsetting studies on CPIs for combinational approaches, perioperative use, new tumor entities, response prediction, toxicity management and use in special patient populations. Further, we identified studies focusing on efficacy and toxicity of anti- CD19 CAR T cells in larger patient cohorts as well as seminal findings on adoptive T cell therapy in other hematological and solid malignancies.

## Checkpoint inhibitors

### Combinational therapy

#### Combination with chemotherapy

Traditionally, chemotherapy and radiotherapy were believed to mediate their anti-cancer effect by direct killing of cancer cells. This concept was challenged over a decade ago by Zitvogel and co-workers who discovered that the antineoplastic effect of chemotherapy, in part, depends on the immunogenic cell death of cancer cells. This leads to immune stimulatory signals via activation of the innate immune system through pattern recognition receptors such as toll-like receptor 4 (TLR4) [5]. Different studies confirmed the immunological effects of chemotherapeutic drugs, in particular, platinum-based agents, and paved the way for the development of combinational regimens using PD-1 / PD-L1-blockade together with established chemotherapeutic drugs [6–11]. Last year saw the completion of several practice-changing phase III trials showing the efficacy of combining PD-1 / PD-L1-blockade with chemotherapy in small cell lung cancer (SCLC), non-small cell lung cancer (NSCLC), HNSCC and breast cancer [12–15]. Currently, more than 170 studies are investigating the promising combination of PD-1/PD-L1 blockade plus chemotherapy in different cancer entities [4].

#### Combination with radiotherapy

Anecdotal reports on systemic anti-tumor response after irradiation of a single tumor lesion date back more than one century [16]. Regression of non-irradiated lesions after localized radiotherapy of a single lesion was first termed ‘abscopal effect’ in 1958 [17]. The underlying mechanism remained unexplained for a long period and it took almost another 50 years, before Demaria et al. concluded that “*Ionizing radiation inhibition of distant untreated tumors (abscopal effect) is immune mediated”* [18]*.* Nowadays, the causative link between local radiation, immunogenic cell death and systemic tumor response is well-established [19]. While the abscopal effect remains a sporadic event, numerous strategies are now under investigation to harness the immunogenic effect of radiotherapy [19].

Given the clinical success of checkpoint blockade, combining radiotherapy with PD-1 / PD-L1 blockade is of special interest. Pre-clinical evidence highlights the synergistic potential of this combination [20]. Translational results from an ongoing phase I/II trial (NCT01976585) investigating local radiotherapy in combination with local application of immunostimulatory agents in patients with indolent lymphoma further support the combination of radiotherapy and PD-1 / PD-L1 blockade [21]. In this trial, patients received 2 Gy of local radiotherapy as part of a so-called “in situ vaccination” (ISV: radiotherapy plus intratumoral application of *Fms*-related tyrosine kinase 3 ligand [Flt3L] and a Toll-like receptor 3 [TLR3] ligand). ISV was able to induce systemic (“abscopal”) tumor regression in three out of eleven treated patients. Importantly, in non-responding patients, the induction of tumor infiltrating PD-1^+^ CD8^+^ T cells was observed, prompting a follow-up trial, which is now recruiting patients for ISV in combination with PD-1 blockade (NCT03789097).

Despite these encouraging findings, negative results for the combination of radiotherapy and checkpoint-blockade have also been recently reported. In a phase II trial in metastatic HNSCC, the addition of local radiotherapy to systemic PD-1 blockade was not able to boost the effect of PD-1-blockade. Here, patients were randomized to receive either nivolumab monotherapy or nivolumab plus stereotactic body radiation therapy (SBRT) of a single tumor lesion. The primary study endpoint - response rate in none-irradiated tumor lesions – was not met. Response rate in patients receiving nivolumab plus SBRT was 22.2% (95% confidence interval [CI]: 10.6–40.8%) versus 26.9% (95% CI: 13.7–46.1%) for single agent nivolumab [22].

The placebo-controlled, randomized phase III PACIFIC trial investigated the addition of durvalumab (anti-PD-L1) to platinum-based chemoradiotherapy in locally advanced (stage III) NSCLC. The addition of durvalumab resulted in an impressive increase in progression-free (PFS) and overall survival (OS) (17.2 versus 5.2 (PFS) and 28.7 months versus “not reached” (OS), respectively) [23, 24]. In this context, the timely administration of PD-1 blockade appeared to be important: patients receiving durvalumab within 14 days after completion of chemoradiotherapy had a better overall survival than patients starting durvalumab-treatment at a later time point [25].

While recent results encourage further in-depth investigation of checkpoint blockade plus radiotherapy, successful concepts might depend on additional combination partners like the above-mentioned in situ-vaccination or chemotherapy. Additional well-designed clinical trials are necessary to identify optimal strategies for combinations and treatment sequences.

#### Combination with immunomodulatory drugs

The first CPI approved for clinical use was ipilimumab, targeting CTLA-4. Given the success of ipilimumab and the even greater success of PD-1-blockade, it is not surprising, that - with more than 250 clinical trials - the combination of PD-1 and CTLA-4 blockade is the most vigorously investigated combinational approach of two immunomodulatory drugs [4].

Due to the large number of clinically approved immunomodulatory agents (currently more than 25) and many more in pre-clinical and clinical development, there is an almost infinite number of combinatorial regimens for further clinical evaluation. In this regard, it is important to note, that the combination of two immunomodulatory drugs can also have antagonistic instead of synergistic effects [26]. Wise selection strategies based on pre-clinical data to select combinatorial approaches for clinical testing are important [26]. In light of this, Tauriello et al. provided an example for an elaborate pre-clinical model system. By using a quadruple mutant colorectal mouse model, they were able to recapitulate important immunological hallmarks of microsatellite stable colorectal cancer (MSS CRC) [27]. While PD-1 / PD-L1 blockade showed only marginal efficacy in this setting paralleling results of clinical trials with PD-1/PD-L1 blockade in MSS CRC, impressive effects were achieved when PD-1/PD-L1 blockade was combined with inhibition of transforming growth factor beta (TGF-β) [27].

Building on pre-clinical and early clinical data for simultaneous targeting of CD40 and PD-1 / PD-L1 in pancreatic cancer (a disease for which all immunotherapeutic efforts have failed so far), a phase I trial investigating the combination of CD40, durvalumab and chemotherapy was initiated. The promising results were recently presented at the annual meeting of the AACR (2019), making this combinational strategy one to keep track of in the years to come [28–30].

### Peri-operative use

Up to now, the clinical use of CPIs has been mainly restricted to advanced tumor stages. Yet, efficacy of checkpoint blockade has been reported to be dependent on baseline tumor burden (with better efficacy observed in patients with low tumor burden), making peri-operative usage of checkpoint blockade an attractive treatment option from a theoretical point of view [31, 32].

Although ipilimumab was approved for the adjuvant treatment of melanoma patients by the FDA (but not by the EMA) based on a placebo-controlled phase III trial reporting superior recurrence-free and overall rates, its use was internationally disputed given the relatively high frequency of serious immune-related adverse events in patients receiving treatment with ipilimumab [33–35]. In Europe, nivolumab was the first checkpoint inhibitor approved for adjuvant treatment of melanoma patients, based on results of the *CheckMate 238* study reported in 2017 [36]. In this study, nivolumab was compared to ipilimumab as adjuvant therapy for patients after resection of stage III-IV melanoma. Recurrence-free survival was reported to be superior while severe adverse events were significantly lower in patients treated with nivolumab (12-month recurrence-free survival: 70.5% vs 60.5%; grade 3 or 4 adverse events: 14.4% versus 45.9% for patients receiving nivolumab or ipilimumab, respectively).

A logical next step to consider would be neoadjuvant use of CPIs. Theoretically, neoadjuvant immunotherapy might be able to prime systemic immunity for tumor surveillance after complete resection – at a time point when tumor antigens are still abundantly present [37]. This concept is supported by recent translational findings from an early clinical study in patients with resectable melanoma: in a randomized phase Ib study, neoadjuvant treatment with nivolumab and ipilimumab induced a higher number of tumor specific T cell clones than adjuvant treatment [38]. Early clinical findings reported from patients with NSCLC, HNSCC and microsatellite unstable (MSI) CRC further emphasize the high potential of neoadjuvant treatment [39–41]. In the latter study, seven out of seven patients with MSI CRC (100%) responded to neoadjuvant treatment with complete remissions observed in 4/7 (57%) patients [41].

A large number of clinical trials is currently investigating neoadjuvant immunotherapy for different disease entities (for example, we identified nine clinical trials for neoadjuvant anti- PD-1 / PD-L1 treatment in NSCLC: NCT03197467, NCT02938624, NCT02259621, NCT03694236, NCT03732664, NCT02994576, NCT03030131, NCT02716038, NCT02818920). Given the considerable side effects of checkpoint blockade – particularly, if administered as combinational therapy - wise selection of patients that might benefit from neoadjuvant or adjuvant treatment is mandatory. One possibility for adjuvant treatment stratification might be detection of minimal residual disease (MRD) by circulating tumor DNA (ctDNA), a strategy, that is currently investigated by a clinical trial in triple-negative breast cancer (TNBC) (NCT03145961) [42].

### New tumor entities

Current studies show the efficacy of CPIs in patients with malignant melanoma (MM), NSCLC or neoplasms with mutational defects in DNA mismatch repair proteins (micro satellite instability or MSI) independent of the actual tumor entity. Intriguingly, all of these tumors share a relatively high mutational load when their genetic characteristics are comparatively analyzed [43]. This common characteristic leads to increased expression of neo antigens in the tumor, stimulating an increased infiltration of the tumor by immune cells, which in turn can be “*activated*” by CPI administration. This fact can also be used to explain why CPI studies in certain tumor entities (among others pancreatic ductal adenocarcinoma (PDAC) or colorectal carcinoma (CRC) without DNA mismatch repair protein defects) haven’t been successful as of yet.

**Fig. 1:**
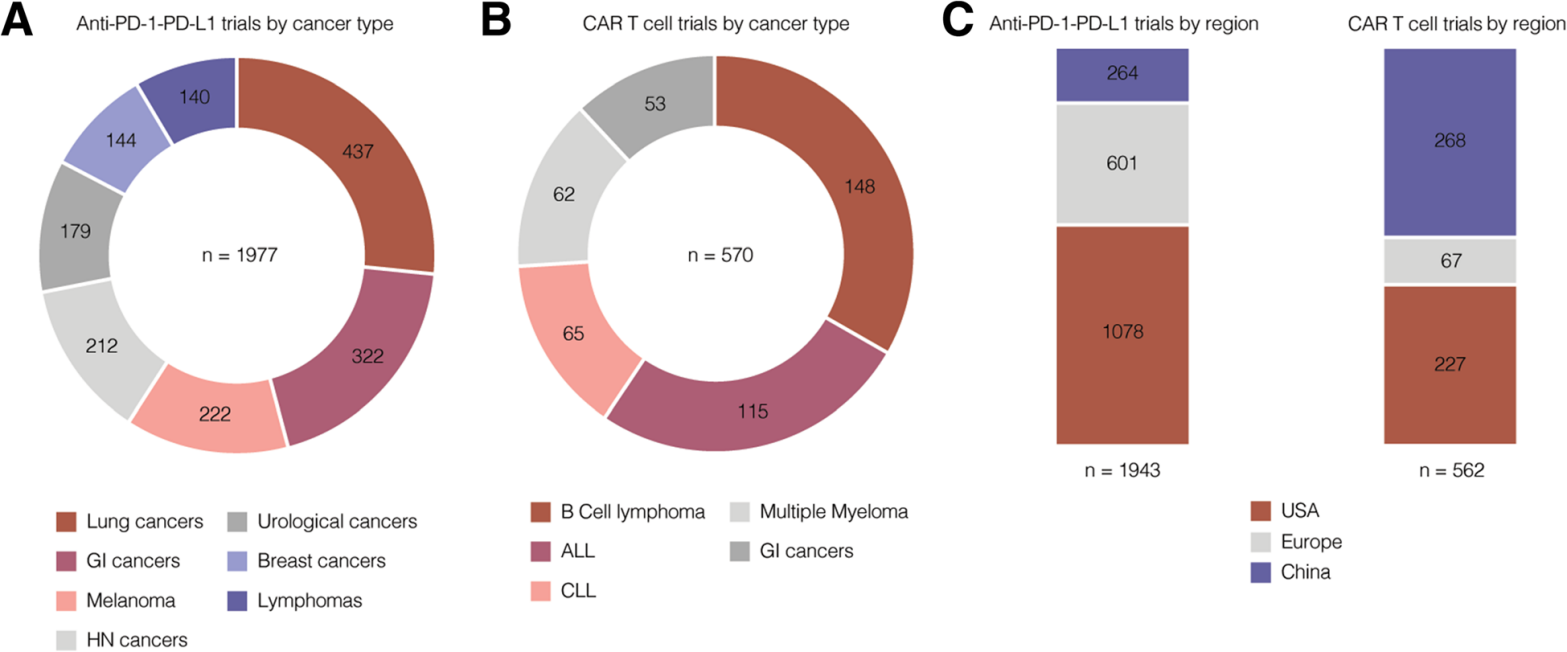
Included tumor types (**a**, **b**) and regional distribution (**c**) of clinical PD-1 / PD-L1 and CAR T cell trials in 2019. ClinicalTrials.gov was searched for “pd-l1” OR “pd-1” OR “programmed death ligand” OR “car t cell” OR “chimeric antigen receptor”. All registered trials were sorted for tumor type and country/region. Search was performed on 2019-05-06. Most frequent tumor types (**a**, **b**) and regions (**c**) are shown as indicated. Several clinical trials included multiple tumor types or were performed in more than one country/region. Abbreviations: GI: gastrointestinal, HN: head and neck

On average, breast cancer and AML are also characterized by a low mutational load [43]. With that background, two remarkable studies from 2018 should be mentioned here in more detail. On the one hand, the phase III trial *IMpassion130* tested the combination of atezolizumab (anti-PD-L1) plus nab-paclitaxel versus nab-paclitaxel monotherapy in treatment-naïve patients with metastatic, triple-negative breast cancer (TNBC). The addition of atezolizumab not only improved the patients’ PFS (PFS), but also their overall survival (OS) [14]. For patients with TNBC, this was the first phase III study that showed a strong benefit of targeted (immune) therapy. A total of 144 studies on PD-1 / PD-L1 blockade in TNBC are currently registered on *clinicaltrials.gov* (Fig. 1a).

On the other hand, for AML, data on nivolumab maintenance therapy in high-risk AML patients was presented at the annual meeting of the American Society of Clinical Oncology (ASCO) in 2018. This study investigated whether the administration of nivolumab might prolong the time of complete remission (CR) in patients that do not qualify for an allogenic stem cell transplantation. In 14 patients that were followed-up for a median of 19.3 months, the median duration of CR averaged 8.3 months, whereas the median OS had not been reached at the time of presentation of the data. Despite the very limited number of patients, this study shows an exciting treatment concept for this specific treatment group [44].

In conclusion, both studies exemplify that successful CPI concepts might also be feasible for tumor entities with a low mutational load. Numerous clinical trials are currently investigating the use of CPIs in different cancer entities (Fig. 1a). It will be interesting to see whether further positive results for tumor entities with low mutational burden will follow in the future.

### Biomarkers for response prediction of checkpoint blockade

Determination of PD-L1 expression by immunohistochemistry is an FDA-approved diagnostic test and a prerequisite for treatment with anti-PD-1 / PD-L1 therapy in various indications (e.g. monotherapy treatment of urothelial cancer with atezolizumab or pembrolizumab). However, determining PD-L1 expression does not identify all patients that profit from anti-PD-1 / PD-L1 therapy, highlighting the need for additional and better biomarkers [45].

#### Tissue biomarkers

##### Microsatellite instability and tumor mutational burden

Another approved biomarker test (for pembrolizumab) is the determination of microsatellite instability (MSI) or deficient mismatch repair (dMMR). Pembrolizumab was the first drug that was FDA-approved with a “tumor-agnostic” indication based on findings from five different clinical trials including 15 tumor entities with MSI/dMMR tumors (KEYNOTE -012, − 016, − 028, − 158 and − 164). MSI/dMMR results in increased tumor mutational burden (TMB) with subsequent increase in neoantigens and immune cell infiltration, rendering tumors susceptible to PD-1 /PD-L1 blockade [46]. In different studies, the direct determination of TMB was also established as predictive biomarker for immunotherapy [47–49]. However, recently presented data suggests that not all patients with MSI/dMMR tumors also have a high TMB [50]. Furthermore, TMB^*high*^ is also observed in the absence of MSI/dMMR [46]. More studies are therefore necessary to inform strategies on selection of MSI/dMMR or TMB as biomarker for response to checkpoint blockade.

##### Tumor mutational burden and PD-L1 expression

It was previously described that TMB does not correlate to PD-L1 expression [51]. This finding was confirmed and put into therapeutic context by the ChekMate227 trial [52]. In this trial, NSCLC patients were stratified according to tumoral PD-L1 expression (≥ 1% vs < 1%). Patients were then randomized (1:1:1) between either chemotherapy, nivolumab (nivolumab plus chemotherapy for patients with < 1% PD-L1 expression, respectively) or nivolumab plus ipilimumab. One predefined endpoint was response rate in patients with a TMB^*high*^ (defined as > 10 mutations per megabase). Independent of PD-L1 expression, nivolumab plus ipilimumab was superior to chemotherapy in patients with high TMB [52].

##### Inflammatory gene signatures

Apart from the biomarkers mentioned above, different inflammatory TMB-signatures determined in tumor tissues can serve as biomarkers for checkpoint blockade. These signatures indicate infiltration by a specific immune cell subset (e.g. effector T cells) or activation of a specific signaling pathway (e.g. interferon-γ signaling). Recently published data from the *IMmotion150* trial suggests that these signatures could even be superior to TMB in patients with metastatic renal cell carcinoma: patients were randomized between the combination of atezolizumab (anti-PD-L1) +/− bevacizumab versus sunitinib. T-effector, interferon-γ and myeloid inflammatory gene expression signatures were superior to TMB in predicting response to atezolizumab [53]. It should be noted, that these analyses were exploratory.

Further research is necessary to integrate the aforementioned tissue biomarkers into one clinical applicable diagnostic algorithm. Well-designed translational studies might also be able to identify completely new tissue biomarkers to predict clinical response to CPI treatment. One example are gene fusions producing immunogenic neoantigens. Such gene fusions were recently shown to predict response to checkpoint blockade in HNSCC patients with low TMB and minimal immune cell infiltrate [54].

#### Soluble biomarkers

Identifying soluble biomarkers for response prediction in peripheral blood would have several advantages over tissue biomarkers. For instance, they are easily and noninvasively accessible and can be sampled repetitively for continuous response prediction. The soluble forms of PD-1 and PD-L1 (sPD1 and sPD-L1) are also present in the peripheral blood [55, 56]. Only few studies have investigated sPD-1 and sPDL-1 as biomarkers for response to checkpoint blockade. One small study in NSCLC patients suggested that high sPD-L1 levels predict poor response to nivolumab [57], a finding that is somewhat contrary to tissue PD-L1, because high PD-L1 tissue expression indicates higher likelihood of response to checkpoint blockade. Findings from patients with pancreatic cancer suggest that sPD-1 and sPD-L1 are rather indicators of systemic inflammation and independent from tumoral PD-L1 expression [56]. Together these findings question the aptitude of sPD-1 and sPD-L1 as biomarkers for checkpoint blockade.

An emerging soluble biomarker for checkpoint blockade is ctDNA in peripheral blood. It can be used for different applications. First, ctDNA can be used to determine tumor mutational burden (TMB) [58]. TMB measured in peripheral blood has been shown to predict response to checkpoint blockade in NSCLC patients [58, 59]. In patients receiving conventional chemotherapy, repeated ctDNA measurement can be used for early response prediction [60]. Recently published studies suggest that changes in ctDNA levels can also be early predictors for response to immunotherapy [61, 62]. Importantly, it might also aid to distinguish pseudo-progression from truly progressive disease in patients treated with immunotherapy [63].

#### Immune related adverse events as biomarker for tumor response

Different studies suggested that immune related adverse events (IrAEs) indicate response to checkpoint blockade [64, 65]. These studies, however, were not controlled for *lead time bias* [66] and it is therefore not clear, whether IrAEs are truly independent predictors for response or merely reflect a longer time under treatment. Recent studies controlled for *lead-time bias* reported conflicting data: a large monocentric study including different cancer types presented at ESMO 2018 did not find a correlation between IrAEs and response to checkpoint blockade after controlling for lead-time bias [67]. Yet, another recent study in renal cell carcinoma reported better efficacy of nivolumab in patients with IrAEs after controlling for lead-time bias [68].

### Toxicity management

#### Use of steroids

The occurrence of immune-mediated side effects (e.g. colitis, autoimmune hepatitis, endocrine or neurological side effects) requires treatment with glucocorticoids (e.g. prednisolone) as early as possible depending on the severity [69]. Whether the use of glucocorticoids has a negative effect on the success of CPI treatment remains controversial. A study presented at the annual meeting of the ASCO in 2018 retrospectively investigated NSCLC patients who received glucocorticoids at the beginning of CPI therapy. The reasons for glucocorticoid administration included the treatment of symptoms caused by brain metastases as well as respiratory distress or fatigue. In a multivariate analysis which included performance status and presence of brain metastases, patients who received glucocorticoids at the start of treatment responded significantly worse to CPI administration [67]. On the other hand, as mentioned in the biomarker section, it is often postulated that patients who develop immune-mediated side effects (and receive glucocorticoids) benefit from CPI therapy over a longer period of time (or at least not shorter) than patients without immune-mediated side effects.

As a practice-based approach, immune-mediated side effects (depending on the severity and type of side effects) should be treated early with glucocorticoids to prevent permanent damage [69]. On the other hand, the need for symptomatic and sustained administration of steroids for other reasons (e.g. brain metastases or respiratory distress) during CPI therapy should be critically scrutinized in everyday clinical practice.

### Special populations: patients with pre-existing autoimmune disease or HIV

Most clinical trials on CPI therapy have excluded patients with pre-existing autoimmune diseases or human immunodeficiency virus infection (HIV). In this regard, it remained unclear whether a CPI therapy is also conceivable in these patients.

The safety and efficacy of CPIs in patients with pre-existing autoimmune diseases has been recently studied in a French registry study including different tumor entities [70]. Patients with and without pre-existing autoimmune diseases were included (patients with pre-existing autoimmune disease: *n* = 45, patients without pre-existing autoimmune disease: *n* = 352). Although the incidence of immune-mediated side effects was significantly increased in the group of patients with pre-existing autoimmune diseases (44% versus 23%), there was no difference in overall survival between the two groups.

For the use of CPIs in patients with HIV, data from a small HIV-positive cohort of patients (*n* = 20) with NSCLC or multiple myeloma was presented at the annual meeting of the European Society of Medical Oncology (ESMO) in 2018. Overall, the therapy with CPIs was well tolerated in patients with HIV and no immune-mediated side effects were observed. An increase in HIV viral load was observed only in one patient who had paused his antiretroviral therapy. A response to therapy (PR or CR) was observed in 24% of patients [71].

Overall, both studies suggest that CPI therapy might be feasible and effective in patients with pre-existing autoimmune disease or HIV. Due to limited data on these special patient groups, a careful assessment of potential benefit versus potential harm is mandatory before starting CPI therapy in these patients.

## Cellular immunotherapy

### Chimeric antigen receptor T cells

*Tisagenlecleucel* and *axicabtagen-ciloleucel* were the first two cellular cancer immunotherapies receiving FDA and EMA approval in 2017 and 2018, respectively. They are approved to treat patients with acute lymphoblastic leukemia (ALL, *tisagenlecleucel*) and diffuse-large B cell lymphoma (DLBCL, *tisagenlecleucel* and *axicabtagen-ciloleucel*). Approval was based on impressive response rates observed in the ELIANA trial (relapsed or refractory [r/r] ALL in pediatric patients or young adults treated with *tisagenlecleucel*), JULIETH trial (r/r DLBCL, *tisagenlecleucel*) and ZUMA-1 trial (r/r DLBCL, *axicabtagen-ciloleucel)* [72–74].

**Fig. 2:**
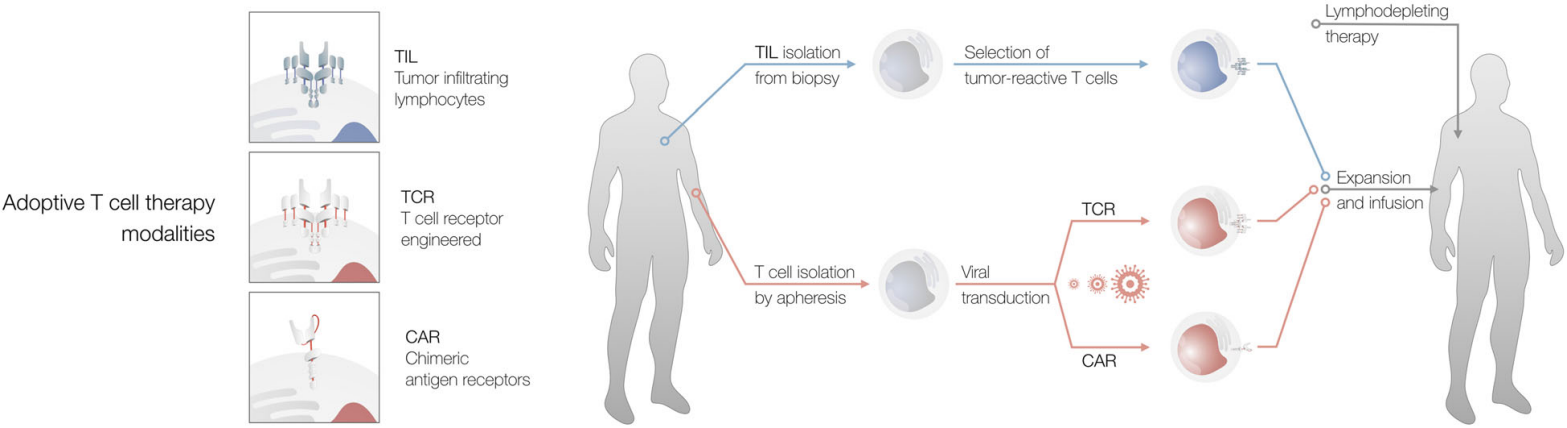
Different strategies for adoptive T cell therapy. Abbreviations: CAR: chimeric antigen receptor, TCR: T cell receptor, TIL: tumor infiltrating lymphocytes

*Tisagenlecleucel* and *axicabtagen-ciloleucel* are autologous T cell products. After leukapheresis, T cells are genetically engineered to express an anti-CD19 chimeric antigen receptor (anti-CD19 CAR T cells). Re-infusion of CAR T cells is preceded by a lympho-depleting chemotherapy to allow for subsequent in vivo expansion of CAR T cells (Fig. 2).

Numerous clinical trials (as of May 2019 more than 550, Fig. 1b) are investigating CAR T cell therapies for different hematological and solid cancer types [75]. Of interest and in harsh contrast to trials on PD-1 / PD-L1 blockade is the regional distribution of clinical trials on CAR T cell therapy (Fig. 1c). The USA and China by far outcompete the EU in terms of registered CAR T cell trials. This regional imbalance has been described and discussed previously and should be addressed by researches and health care policy makers in the European Union [76].

Recently reported studies on cellular therapy mainly addressed two important questions: (I) Long term and “real world” experience regarding toxicity and efficacy of CAR T cells (II) Can the striking success of CAR T cells in ALL and DLBCL be translated to other hematological and – more importantly - solid malignancies?

#### Updated results from CD19 CAR T cells clinical trials

Follow-up results for efficacy and toxicity from the ELIANA, JULIETH and ZUMA-1 trial were recently presented at the annual meetings of the European Hematology Association (EHA) and the American Society of Hematology (ASH).

##### Efficacy

As of 2018, 97 patients aged ≤21 years with r/r ALL were enrolled in the ELIANA trial, 79 patients were infused with CD19 CAR T cells and a complete remission was achieved in 65 patients. After a median follow-up of 24 months, response was ongoing in 29 patients (45%), with a maximum (ongoing) duration of response of 29 months [77]. For r/r DLBCL patients treated with *tisagenlecleucel,* the updated analysis presented at EHA 2018 included 111 infused patients. Overall response rate (ORR) was 52% (40% CR, 12% PR) [78]. After a median follow-up time of 14 months, median duration of response was not reached. Median overall survival for all infused patients was 11.7 months [79]. For *axicabtagen-ciloleucel,* the 2-year follow-up data was presented at ASH 2018. A total of 108 r/r DLBCL patients had at least one year of follow-up. ORR in this cohort was 82% (58% CR). An ongoing response was observed in 42% of all patients after a median follow-up of 15.4 months, no updated overall survival data was reported [80].

For *axicabtagen-ciloleucel, “real world”* efficacy was confirmed by data from seventeen US academic centers who evaluated *axicabtagen-ciloleucel* outside of clinical trials, independent of the manufacturer after commercialization. The authors reported an ORR of 79% (50% CR), confirming the results reported in the clinical trials mentioned above [81].

While these results support the high therapeutic potential of CAR T cell therapy, a cohort of patients does not respond to – or relapses after – CAR T cell therapy. Considering the latter group (relapse after an initial complete response), it is important to explore further treatment options for these patients. One possibility might be allogeneic stem cell transplantation, which has recently been reported to improve prognosis after anti-CD19 CAR T cell therapy for ALL patients who had not received a previous stem cell transplantation [82].

##### Toxicity

The updated data for ELIANA, JULIETH and ZUMA-1 confirm the previously described safety profile with cytokine release syndrome (CRS, incidence of CRS grade ≥ 3: 7 to 48%) and neurologic events (NE, incidence of NE grade ≥ 3: 11 to 31%) as most significant adverse events [78–81].

In the pivotal trials for anti-CD19 CAR T cells, treatment-related deaths have been reported [77]. No treatment-related deaths were observed in a US multi-center cohort of 165 patients who received *axicabtagen-ciloleucel* for r/r DLBCL after commercialization outside of clinical trials [81]. Recently, safety of *axicabtagen-ciloleucel w*as also confirmed in patients ≥65 years [83]. Further it has been reported that neurotoxicity is fully reversible in most patients [84].

While the mentioned results are reassuring regarding saftey of CAR T cell therapy, different strategies are currently under investigation to further improve the safety profile of CAR T cells. These strategies include: (I) modification of the chimeric antigen receptor cell itself [85, 86]; (II) identification of predictive biomarkers for CAR T cell toxicity [84]; (III) “safety switches” such as inducible suicide genes [87]; and (IV) novel drugs to mitigate CRS and NE [88].

#### Adoptive T cell therapy in other hematological and solid malignancies

##### Chimeric antigen receptor T cells for hematological and solid malignancies

The success of CAR T cells in ALL and B cell lymphoma led to the initiation of numerous follow-up trials in these disease entities (Fig. 1b). Regarding other cancer types, chronic lymphocytic leukemia, multiple myeloma and gastrointestinal cancers are the ones with most clinical CAR T cell trials underway (Fig. 1b).

Additionally, a large variety of strategies to improve efficacy of CAR T cells in solid malignancies are under pre-clinical investigation [89–94]. Yet, the direct translation of the CAR T cell approach to solid malignancies is often impeded by the lack of a suitable cancer specific antigen resulting in either disappointing efficacy or substantial off target toxicity in early clinical trials [95]. Another important consideration is the tumor environment which is substantially different to the one seen in the above referenced hematological cancers and impedes CAR T cell efficacy [96].

Alternative approaches are genetic modification of the T cell receptor (TCR) itself or the adoptive transfer of “naturally” occurring tumor reactive T cells (also termed tumor infiltrating lymphocytes or TILs) isolated from autologous tumor tissue or tumor draining lymph nodes (Fig. 2). The manufacturing of TCR-modified T cells is complex, dependent on a specific human leukocyte antigen (HLA)-haplotype and can lead to unexpected off-target toxicity [97, 98]. On the other hand, the use of tumor reactive (TCR-native) T cells has been investigated in numerous clinical studies (mainly in melanoma patients) with promising results [99, 100]. Recent studies suggest that this approach could also be successfully translated to other solid malignancies.

##### Ex vivo *expansion and reinfusion of autologous tumor reactive T cells*

In contrast to CAR T cells, tumor reactive T cells recognize tumor cells via their native (unmodified) T cell receptor (Fig. 2). Tumor reactive T cells can be isolated from tumor tissue or tumor draining lymph nodes [101–106]. After a potential selection step followed by ex vivo expansion, tumor reactive T cells are re-infused after lymphodepleting chemotherapy – typically with parallel intravenous administration of interleukin 2 [101]. The high potential of this approach was recently confirmed in melanoma patients after failure of PD-1 / PD-L1 blockade [107] and is currently investigated in a phase III trial as first-line treatment for advanced melanoma patients (NCT02278887). In other solid tumor entities an ongoing early clinical trial (NCT01174121) is currently investigating immunotherapy with tumor reactive T cells in patients with metastatic gastrointestinal, urothelial, breast, ovarian or endometrial cancer. Case reports from three individual patients described striking responses for this treatment approach for cholangiocarcinoma, colorectal cancer and breast cancer, respectively [104–106]. Further studies are necessary to evaluate the expansion of this promising treatment approach to larger patient populations.

## Conclusion

Immunotherapy of cancer is a rapidly evolving field. Results of currently ongoing studies on checkpoint blockade will most likely expand the use of CPIs to additional patient populations (e.g. new tumor entities, perioperative use, use in special patient populations) and might identify new combination partners for CPI.

The major challenge for adoptive T cell therapy in years to come is the translation of this treatment modality to solid malignancies. A successful strategy has yet to be defined and might include more advanced genetic engineering of CAR T cells as well as the development of more advanced protocols for the use of tumor reactive (TCR-native) T cells.

Regarding the regional distribution of clinical trials on immunotherapy a shift from the European region (for PD-1 / PD-L1-trials) towards China (leading in terms of number of available CAR T cell trials) is evident and should be met by intensified research efforts on cellular immunotherapy in Europe.

## Data Availability

The datasets generated and analysed for Fig. 1 are available in the U.S. National Library of Medicine repository, https://clinicaltrials.gov/

## Abbreviations

ALL: Acute lymphoblastic leukemia
ASCO: American Society of Clinical Oncology
CAR T cells: Chimeric antigen receptor T cells
CPIs: Checkpoint inhibitors
CR: Complete remission
CRC: Colorectal cancer
CRS: Cytokine release syndrome
ctDNA: Circulating tumor DNA
CTLA-4: Cytotoxic T-lymphocyte-associated protein-4
DLBCL: Diffuse-large B cell lymphoma
dMMR: Deficient mismatch repair
EHA: European Hematology Association
EMA: European medicines agency
ESMO: European Society of Medical Oncology
FDA: U. S. food and drug administration
GMP: Good manufacturing practice
HIV: Human immunodeficiency virus
HNSCC: Head and neck squamous cell carcinoma
IrAEs: Immune related adverse events
MSI: Microsatellite unstable
MSS: Microsatellite stable
NE: Neurologic events
NSCLC: Non-small cell lung cancer
ORR: Overall response rate
OS: Overall survival
PD-1: Programmed cell death protein 1
PDAC: Pancreatic ductal adenocarcinoma
PD-L1: Programmed cell death protein ligand 1
PFS: Progression free survival
PR: Partial remission
r/r: Relapsed or refractory
RCC: Renal cell cancer
RNA: Ribonucleic acid
SBRT: Stereotactic body radiation therapy
SCLC: Small cell lung cancer
sPD1: Soluble form of PD-1
sPD-L1: Soluble form of PD-L1
TCR: T cell receptor
TLR4: Toll-like receptor 4
TMB: Tumor mutational burden
